# Injuries to the anterolateral ligament are observed more frequently compared to lesions to the deep iliotibial tract (Kaplan fibers) in anterior cruciate ligamant deficient knees using magnetic resonance imaging

**DOI:** 10.1007/s00167-021-06535-6

**Published:** 2021-03-26

**Authors:** Armin Runer, Dietmar Dammerer, Christoph Kranewitter, Johannes M. Giesinger, Benjamin Henninger, Michael T. Hirschmann, Michael C. Liebensteiner

**Affiliations:** 1 Department of Orthopaedics and Traumatology, Medical University of Innsbruck, Anichstrasse 35, 6020 Innsbruck, Austria; 2 Department of Orthopaedic Surgery, Medical University Innsbruck, Innsbruck, Austria; 3 Department of Radiology, Medical University Innsbruck, Innsbruck, Austria; 4 Innsbruck Institute of Patient-Centered Outcome Research (IIPCOR), Innsbruck, Austria; 5 Department of Orthopaedic Surgery and Traumatology, Kantonsspital Baselland (Bruderholz, Liestal, Laufen), Bruderholz, Switzerland

**Keywords:** Anterolateral ligament, ALL, Iliotibial tract, Iliotibial band Kaplan fibers, Anterior cruciate ligament rupture, ACL, MRI, Anterolateral knee complex, Knee, Intrarater reliability, Interrater reliability

## Abstract

**Purpose:**

To determine the accuracy of detection, injury rate and inter- and intrarater reproducibility in visualizing lesions to the anterolateral ligament (ALL) and the deep portion of the iliotibial tract (dITT) in anterior cruciate ligament (ACL) deficient knees.

**Methods:**

Ninety-one consecutive patients, out of those 25 children (age 14.3 ± 3.5 years), with diagnosed ACL tears were included. Two musculoskeletal radiologists retrospectively reviewed MRI data focusing on accuracy of detection and potential injuries to the ALL or dITT. Lesion were diagnosed in case of discontinued fibers in combination with intra- or peri-ligamentous edema and graded as intact, partial or complete tears. Cohen’s Kappa and 95% confidence intervals (95% CI) were determined for inter- and intrarater reliability measures.

**Results:**

The ALL and dITT were visible in 52 (78.8%) and 56 (84.8%) of adult-and 25 (100%) and 19 (76.0%) of pediatric patients, respectively. The ALL was injured in 45 (58.5%; partial: 36.4%, compleate: 22.1%) patients. Partial and comleate tears, where visualized in 21 (40.4%) and 16 (30.8%) adult- and seven (28.0%) and one (4%) peditric patients. A total of 16 (21.3%; partial: 13.3%, compleate: 8.0%) dITT injuries were identified. Partal and complete lesions were seen in seven (12.5%) and five (8.9%) adult- and three (15.8%) and one (5.3%) pediatric patients. Combined injuries were visualized in nine (12.7%) patients. Inter-observer (0.91–0.95) and intra-observer (0.93–0.95) reproducibility was high.

**Conclusion:**

In ACL injured knees, tears of the ALL are observed more frequently compared to lesions to the deep iliotibial tract. Combined injuries of both structures are rare. Clinically, the preoperative visualization of potentially injured structures of the anterolateral knee is crucial and is important for a more personalized preoperative planning and tailored anatomical reconstruction. The clinical implication of injuries to the anterolateral complex of the knee needs further investigation.

**Level of evidence:**

II.

## Introduction

Recently, there has been an increased interest in the anterolateral extra-articular soft-tissue structures of the knee. In particular the anterolateral ligament (ALL) was popularized as an important stabilizer [5]. This triggered several investigations either on traceability of the ALL during cadaver dissection [5, 7, 12, 37, 39, 40, 47], the biomechanical characteristics [23, 33, 37, 41, 46] or the visibility of the ALL using magnetic resonance imaging (MRI) [11, 13–15, 25, 26, 30, 38, 48].

Several previous studies dealt with MRI—visibility of the ALL both in healthy and ACL injured knees [11, 13–15, 25, 26, 30, 38, 48]. A recent systematic review reported a high variability in visualizing the intact ALL (51–100%) as well as identifiying potential injuries to the structure (10.7–98%) when using MRI [1].

While most of the above-mentioned authors promote the ALL as a main anterolateral knee stabilizer opposing excessive internal tibial rotation and subluxation, others doubt the relevance of the ALL in providing anterolateral knee stability, putting more emphasis on the role of the deep portions of the iliotibial tract, including the so called Kaplan fiber complex [18, 22, 35]. The deep portion of the iliotibial tract (dITT) was first described in 1958 by Kaplan et al. and consists of two distinct parts: the deep attachments of the ITT to the distal femur (Kaplan fibers, KF) and the capsulo-osseous layer of the ITT (COL) [20, 29, 34, 49]. The Kaplan fibers have been further distinguished in proximal and distal supracondylar fibers connecting the ITT to the postero-lateral femur approximately 28–41 mm above the lateral femoral epicondyle [16, 17, 29, 42]. Recent articles examining both healthy individuals and knee specimens reported good MRI visibility of the dITT ranging between 60.6 and 100% [2, 3, 28, 32].

Up to date, no study has yet investigated the variability and reliability in diagnosing lesions to both the ALL and the dITT in adult and pediatric patients with ACL deficient knees.

The aim of the present study was to answer the following study questions: (1) What is the visibility of the ALL and dITT in adult and pediatic ACL deficient knees using MRI? (2) What is the injury rate of the ALL and the dITT in adult and pediatic ACL deficient knees? (3) What is the inter- and intra-observer reproducibility in detecting lesions to the ALL and dITT in ACL deficient knees using MRI?

## Materials and methods

Ethical approval was obtained from the ethical committee of the Medical University of Innsbruck (AN20 15-0269 355/4.18). The analysis was conducted in accordance with the ethical standards of the Declaration of Helsinki.

### Patients

MRI data of 91 consecutive sujects, out of those 25 children, with clinical and radiological diagnosed ACL rupture were retrospectively reviewed focusing on the accuracy of detection and potential injuries to the ALL or dITT. All diagnoses were made by sports-medicine trained orthopaedic surgeons and two musculosceletal radiologists, respectively. Prior to inclusion an in depth clinical history was obtained and medical records were checked for previous knee injuries or surgeries. Patients were excluded in case of metallic material such as screws or plates around the knee, motion artifacts or different MRI protocols.

### Radiological analysis

All MRIs were obtained using an identical protocol. Patients were examined in supine position with extended knees using a dedicated 15-channel knee coil. The following sequences were used for the 3.0 T Scanner (Avanto/Skyra, Siemens, Erlangen, Germany): coronal T1-weighted images (TE 10/13, TR 696/522, SL 3 mm); coronal PD-weighted images with fat-saturation (TE 40/38, TR 4100/3230, SL 3 mm); sagittal PD-weighted images with fat-saturation (TE 39/38, TR 3000/3710, SL 3 mm) and axial PD-weighted images with fat-saturation (TE 31/37, TR 3010/3100, SL 3.5/3 mm). Two trained and certified musculoskeletal radiologists (HB, KC) with dedicated MRI experience analyzed coronal, sagittal and axial MRI sets of all patients using the imaging viewer Impax EE (Agfa Health Care N.V., Mortsel, Belgium). Before the start of the analysis, a specialist in the field of anterolateral knee anatomy lectured and briefed both radiologists in a private cadaver dissection classe All relevant lateral and anterolateral structures of the knee were dissected and studied.

Both radiologist were blinded to the clinical findings of the patients. Each radiologist performed the analysis twice with an interval in between greater than 2 weeks. For ALL diagnostic, a low-signal ligamentous structure originating from the postero-proximal region of the lateral femoral epicondyle, running in an anterodistal direction deep to the ITT, crossing the lateral collateral ligament in the proximal third and inserting on the anterlateral tibia between Gerdy’s tubercle and the fibular head, was searched. For the diagnostic of the dITT an extraarticular, low-signal band structure connecting the ITT to the proximal ridge of the distal femur was scouted. Detailed definition of both structures as well as diagnostic criteria for MRI identification are reported in Table 1.

**Table 1:** Diagnostic criteria for MRI identification of the ALL and deep portion of the ITT [9]

Anterolateral Ligament (ALL)
Low-signal band on PD sequences
Seen on two sequences under cross-reference
Extra-articular structure
Origin at the postero-proximal region of the femoral epicondyle
Running in an antero-distal direction deep to the ITT
Crossing the lateral collateral ligament in its proximal third
Inserting on the anterolateral tibia midway between Gerdy’s tubercle and the fibular head
Deep portion of the ITT (dITT)
Kaplan fiber complex
Low signal band on PD sequences
Seen on two sequences under cross-reference
Extra-articular structure
Running postero-lateral and continuing distally from the intermuscular septum
Fibers connecting the ITT to the proximal ridge of the distal femur
Seen on 2 consecutive slices
Capsulo-osseous layer
Low signal band on PD sequences
Seen on two sequences under cross-reference
Fibers starting from the region of the Kaplan fiber complex
Running to the anterolateral tibia

*ALL* Anterolateral Ligament, *ITT* Iliotibial Tract, *PD* proton density

Each radiologiest categorized both structures first as either visible or non visible and subsequentely as intact, partial or complete injured. A complete ALL or dITT lesion was diagnosed in case of discontinued fibers in combination with intra- or peri-ligamentous edema. A partial ruptur was defined as an alteration of the normal fiber contiguity but without a clear ligament disruption. Abnormal intraligamentous signal and surrounding periligamentous edema may be present in partial ligament ruptures. In case an ALL injury was diagnosed, further subspecification in proximal- (above meniscus level) and distal- (below meniscus level) intraligamentous lesions as well as tibial avulsion fractures (Segond type) were made. For ITT lesions, a further subclassification in (a) proximal lesions of the deep ITT (Kaplan fiber complex) or (b) lesions to the COL of the ITT [20, 29, 34, 49] was conducted. The COL was defined according to previous recommendations as deep fibers running from the region of the Kaplan fiber complex to the anterolateral tibia [9, 20, 29, 34, 49]. After data analysis and inter-and intra-observer calculation, discrepant radiological findings were reviewed, discussed and a consensus was found between both radiologists. The results of the consensus are reported in the result section and Table 3.

### Statistical analysis

Descriptive statistics and statistical analysis were performed using SPSS v. 22 (IBM Corp.). Cohen’s Kappa and 95% confidence intervals (95% CI) were determined as a measure of inter- and intra-observer reproducibility. A value of 0.70 for Cohen’s Kappa was considered as threshold for substantial reproducibility [27]. Sample size considerations were based on power analysis for a Pearson correlation as an approximation for the Cohen’s Kappa coefficient. Power analysis for Cohen’s Kappa was not available in common power analysis software packages. An observed correlation coefficient of 0.83 in a sample of 60 cases was sufficient to demonstrate exceedance of the 0.70 threshold with alpha = 0.05 and beta = 0.20 (one-sided). Power analysis was done with G*Power 3.1.9.2 [8]. The following scale of measurement agreement was applied: 0–0.2, slight; 0.21–0.4, fair; 0.41–0.6, moderate; 0.61–0.8, substantial; 0.81–1, almost perfect [27].

## Results

A total of 66 adult (35 female) and 25 children (10 female) patients with clinical and radiological diagnosed complete ACL ruptures were included in the study. Patient demographics and additional injuries are reported in Table 2.

**Table 2:** Patients demographics

	Adult patients	Pediatric patient	Total
*N*	66	25	91
Gender (m/f)	31/35	15/10	46/45
Age (mean ± SD)	38.4 ± 14.1	14.3 ± 3.5	31.8 ± 16.3
BMI	27.1 ± 19.8	27.1 ± 15.5	27.1 ± 18.7
Additional injuries^a^			
Medial meniscus rupture	28	8	34
Lateral meniscus rupture	11	3	14
MCL injury	24	7	31
LCL injury	9	2	11
Chondromalacia	20	0	20
PCL injury	0	2	2

*m/f* male/female, *BMI* body mass index, *MRI* magnetic resonance imaging, *MCL* medial collateral ligament, *LCL* lateral collateral ligament, *PCL* posterior cruciate ligament

^a^Injuries as radiological reported

### Visibility

In 77 (84.6%) of all patients the ALL was visible. The deep femoral attachments of the dITT (Kaplan fiber complex) were identified in 75 (82.4%) of the cases. The COL of the ITT was not seen in any patient. For further subclassification in adult and pediatric patients see Table 3.

**Table 3:** Visibility and rupture frequencies for the anterolateral ligament (ALL) and the deep portion of the iliotibial tract (dITT) in adult and pediatric patients with ACL deficiency

	Adult patients (*n* = 66)			Pediatric patients (*n* = 25)			Total patients (*n* = 91)		
	Visible *n* (%)	Complete tear *n* (%)	Partial tear *n* (%)	Visible *n* (%)	Complete tear *n* (%)	Partial tear *n* (%)	Visible *n* (%)	Complete tear *n* (%)	Partial tear *n* (%)
Anterolateral ligament^a^									
Proximal part	60 (90.9)	10 (16.7)	19 (31.7)	25 (100)	1 (4)	4 (16)	85 (93.4)	11 (12.9)	23 (27.1)
Distal part	55 (83.3)	13 (23.6)	7 (12.7)	25 (100)	0 (0)	4 (16)	80 (87.9)	13 (16.3)	11 (13.8)
Tibial avulsion (Segond)	66 (100)	0 (0)	0 (0)	25 (100)	1 (4)	0 (0)	91 (100)	1 (1.1)	0 (0)
Illiotibial tract^a^									
Deep femoral attachment (KFC)	56 (84.8)	5 (8.9)	7 (12.5)	19 (76.0)	1 (5.3)	3 (15.8)	75 (82.4)	6 (8.0)	10 (13.3)
Capsulo-osseous layer	n.a	n.a	n.a	n.a	n.a	n.a	n.a	n.a	n.a
Anterolateral ligament (any part)^a^	52 (78.8)	16 (30.8)	21 (40.4)	25 (100)	1 (4)	7 (28.0)	77 (84.6)	17 (22.1)	28 (36.4)
Iliotibial tract (any part)^a^	56 (84.8)	5 (8.9)	7 (12.5)	19 (76.0)	1 (5.3)	3 (15.8)	75 (82.4)	6 (8.0)	10 (13.3)
Torn ALL (any part) and dITT (any part)^a,b^	52 (78.8)	2 (3.8)	5 (9.6)	19 (76.0)	0.0)	2 (10.5)	71 (78.0)	2 (2.8)	7 (9.9)

Percentages of tear frequencies are reported. The absolute numbers are reported in relation to the number of visible cases [excluding "Tibial avulsion (Segond)]

*n.a.* not applicable, *ALL* anterolateral ligament, *dITT* deep portion of the Iliotibial Tract, *KF* Kaplan fiber complex

^a^Displayed as number and percentage; ^b^applicable, if in both anatomical structures (ALL and dITT) at least one part is injured

### Injury rate

**Fig. 1:**
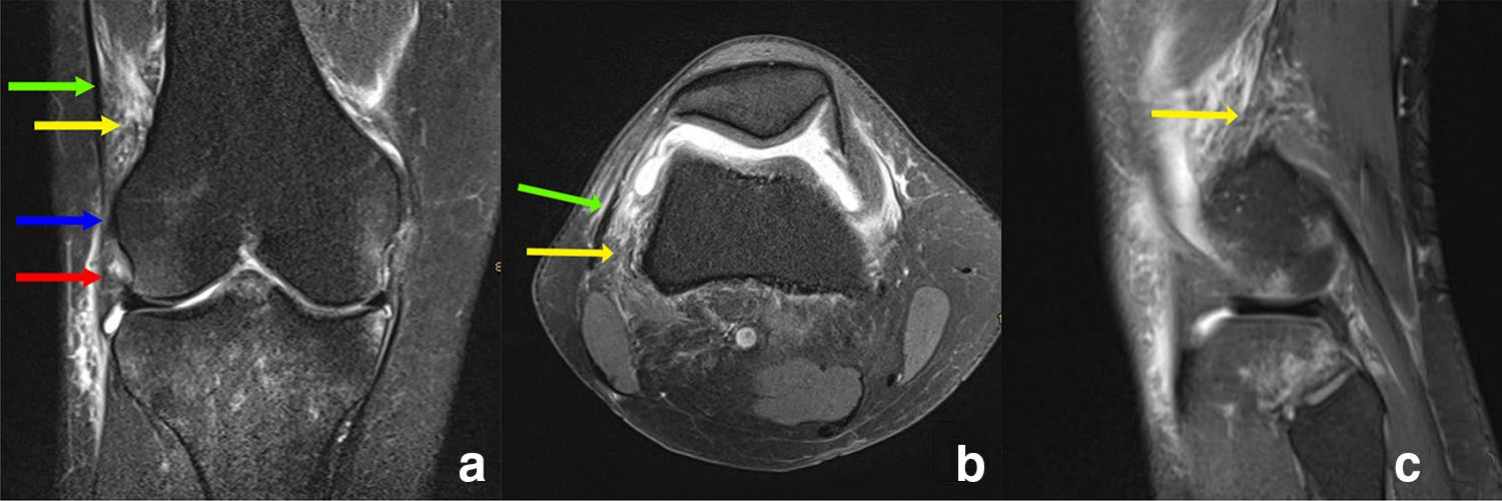
Coronal (**a**), axial (**b**) and sagittal (**c**) PD-weighted fat-saturated image with a suprameniscal tear of the ALL (red arrow) and a complete tear of the Kaplan fiber complex (yellow arrow) indicated by the wavy appearance and surrounding edema green arrow = superficial ITT; blue arrow = lateral collateral ligament

**Fig. 2:**
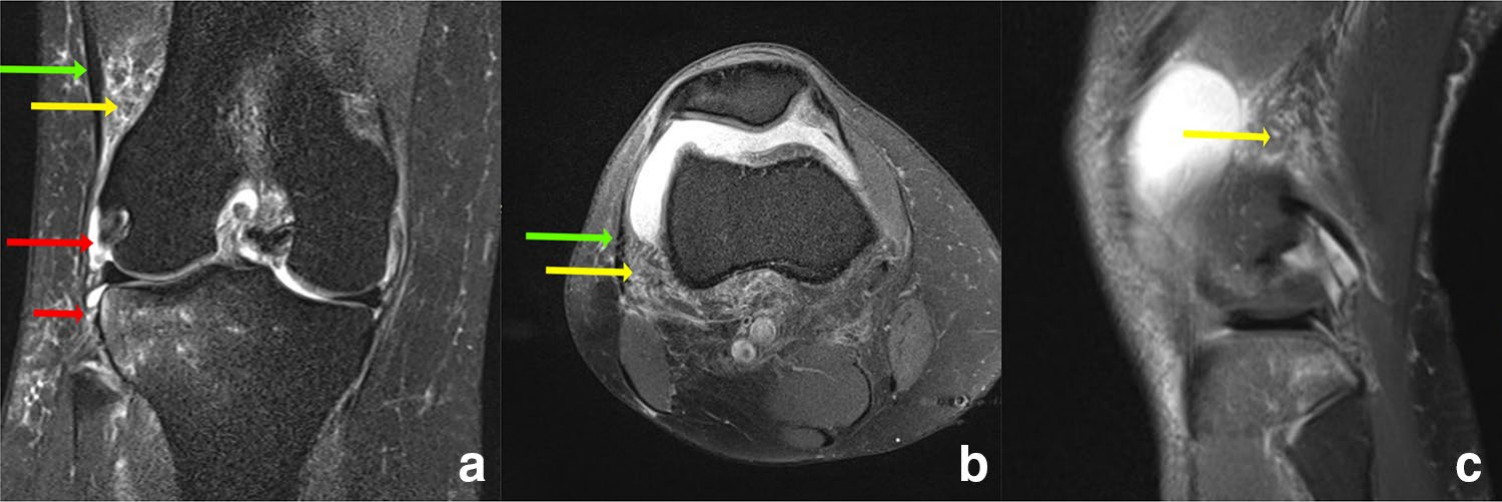
Coronal (**a**), axial (**b**) and sagittal (**c**) PD-weighted fat-saturated image with a complete lesion of the Kaplan fiber complex (yellow arrow) and complete tear of the suprameniscal (long red arrow) and inframeniscal (short red arrow) ALL. There is diffuse edema around the Kaplan fiber complex green arrow = superficial ITT

**Fig. 3:**
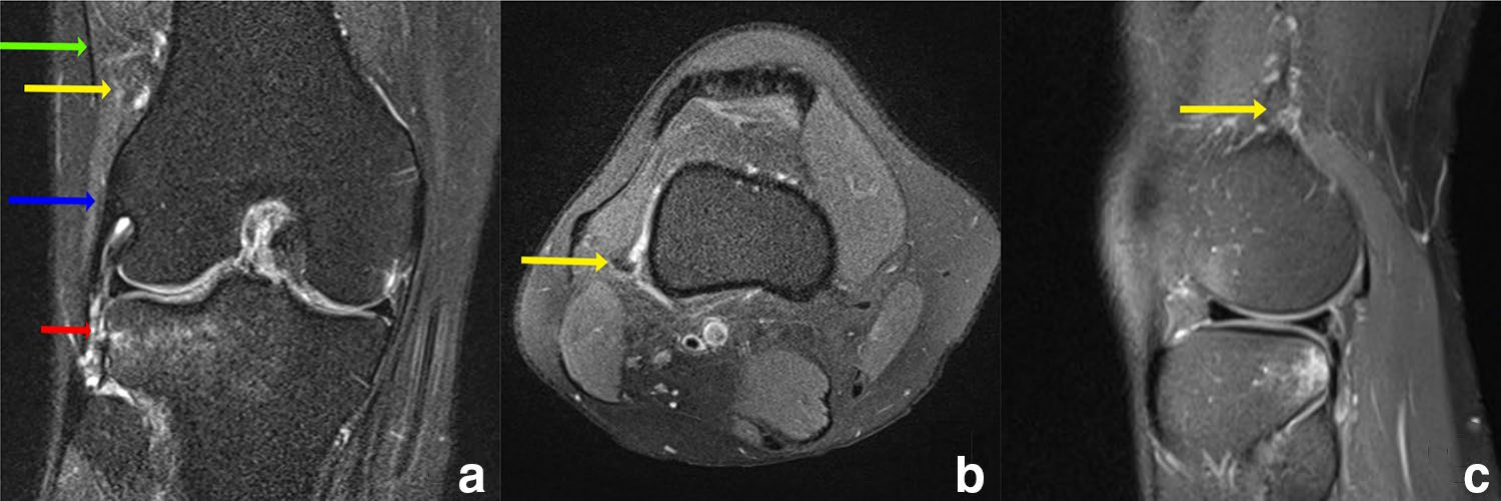
Coronal (**a**), axial (**b**) and sagittal (**c**) PD-weighted fat-saturated image with a lesion to the inframeniscal ALL (short red arrow) but intact Kaplan fiber complex (yellow arrow). There is diffuse edema around the ALL, while no signal alteration is visible next to the Kaplan fiber complex green arrow = superficial ITT; blue arrow = lateral collateral ligament

An injury to the ALL as seen in 45 (58.5%) of the patients. Out of those were 17 (22.1%) complete- and 28 (36.4%) partial ALL tears, respectively. In none of the cases a tibial avulsion (Segond lesion) was detected. An lesion to the dITT was visualized in 16 (21.3%) of the patients with six (8.0%) beeing complete and 10 (13.3%) beeing partial ruptures. Combined injuries of both the ALL and the dITT was reported in nine (12.7%) patients. For further subclassification in adult and pediatric patients see Table 2. Examples of intact and ruptured structures are presented in Figs. 1, 2 and 3.

### Reliability analysis

Detailed inter- and intra-observer data are presented in Table 4. Overall inter-observer agreement was high with calculated Cohen’s Kappa values of 0.95 (95% CI 0.89–1.00) for proximal ALL injuries, 0.91 (95% CI 0.82–0.99) for distal ALL lesions and 0.94 (95% CI 0.86–1.00) for ruptures of the Kaplan fiber complex. The COL was not visible in any patients. For each of the investigated parameters absolute agreement between the observers was at least 94.0%. Calculated intra-observer reliability values were 0.95 (95% CI 0.89–1.00) for proximal ALL lesions, 0.93 (95% CI 0.85–1.00) for distal ALL lesions and 0.94 (95% CI 0.87–1.00) for lesions of the Kaplan fibers complex. For each of the investigated parameters absolute agreement between the two time points was at least 0.95.

**Table 4:** Inter- and intra-observer reproducibility results for the tear rates of the ALL and the dITT

	Interobserver reliability		Intraobserver reliability	
	Cohen's Kappa (95% CI)	Absolute agreement (%)	Cohen's Kappa (95% CI)	Absolute agreement (%)
Anterolateral ligament				
Proximal part	0.95 (0.89–1.00)	97.1	0.95 (0.89–1.00)	97.1
Distal part of ALL	0.91 (0.82–0.99)	94.0	0.93 (0.85–1.00)	95.5
Tibial avulsion (Segond)	n.c	100	n.c	100
Iliotibial tract				
Deep femoral attachments (Kaplan fibers)	0.94 (0.86–1.00)	97.1	0.94 (0.87–1.00)	97.1
Capsulo-osseous layer	n.c	100	n.c	100

Inter- and intra-observer reproducibility results for the different parts of the anterolateral ligament (ALL) (proximal, distal tibial avulsion) and the deep iliotibial tract (ITT) structures (deep attachments of the ITT to the distal femur, capsulo-osseous layer of the ITT)

*95% CI* 95% confidence interval, *n.c.* not calculated

## Discussion

The most important finding of the present study was, that tears of the ALL (58.5%) are observed more frequently compared to lesions to the dITT (21.3%) in ACL deficient knees. Combined injuries of both structures are rare (12.7%). Both the ALL and dITT can be asses with high inter- and intra-observer reliability when using MRI.

Poor clinical outcomes, functional knee instability and an increased risk for osteoarthritis due to persistent anterolateral rotatory instability (ALRI) after ACL reconstruction led to an increased interest in the anterolateral knee structures, predominantly to the ALL and the deep portion of the ITT. However, biomechanical results are inconsistent whether one or the other structure provides more anterolateral stability to the knee joint [6, 9, 22, 24, 35, 36, 43, 44]. A fact, which is also reflected in the number of available different extra-articular reconstruction techniques to address high-grade rotatory instability. While some authors advocate ACL surgery in combination with an anatomical ALL reconstruction [19, 45], others prefer a lateral tenodesis by inserting the graft more proximal at the femur, mimicking the deep structures of the ITT rather than the ALL [4, 31].

Contradictory knowledge exists about the true injury rates as well as the validity and reproducibility in assessing lesions to the ALL and the dITT using MRI. A more profound radiological understanding, however, is crucial for a more personalized preoperative planing and more tailored anatomical reconstruction of these structures.

Regarding the ALL, an increased but heterogeneous body of evidence has emerged lately with tear rates ranging between 10.7 and 98% in ACL deficient knees [1]. Intra- and inter-observer reliabilities vary between 0.04–0.86 and 0.33–1.0, respectively [1]. The present findings of 22.1% complete- and 36.4% partial ALL lesions in patients with ACL rupture are in the middle of the injury ranges stated in the literature. Contrary, intra- and intra-rater reliabilities were rather high, ranging between 95.5–97.1 and 94.0–97.1, respectively. These high agreements within and between both rather might be best explained by the high experience level of both fellowship-trained musculoskeletal radiologist and the usage of a modern 3 T scanner with a dedicated 15-channel knee coil.

Besides numerous studies analyzing the ALL, little knowledge exists about the injury incidence of the deep portion of the ITT. This is somewhat surprising, since those structures have been described anatomically much earlier than the ALL, are believed to work as an agonist to the ACL (like a horseshoe) and are emphasized by some authors to play a more important role than the ALL in providing anterolateral rotatory stability [10, 20, 22, 29, 34, 49]. Batty et al. [2] examined 50 healthy knees using MRI and identified the dITT in 96% of the cases on sagittal images and 76% in the axial view. Inter-observer reliability assessment revealed slight to moderate agreement with Kappa values ranging between 0.1 and 0.5. In a recently published study of our study group, the Kaplan fiber complex could be visualized in 60.6% of healthy knees with high inter- and intra-observer reliabilities [28]. Berthold et al. identified the proximal and distal fibers of the ITT in 100% and 90% of specimen knees using MRI and in 100% of cadaver dissection [3].

Little and contrary evidence exists about the incidence rate of dITT injuries in ACL deficient patiens (Table 5). Van Dyck et al. [50] examined 69 patients reporting assosciated injuries to the Kaplan fibers in 33% (30% periligamentous edema, 3% partial tear, 0% compleate tear) and injuries to the ALL in 57% of the patients (17% periligamentous edema, 32% patial tear, 7% compleate tear). Khanna et al. [21] reported in a cohort of 20 patients an injury rate of 82% to the proximal- and 29% to the distal fibers, respectively. Recently, Marom et al. [32] identiefied with moderate to good inter- and intrarater reliablity injuries to the Kaplan fiber complex in 51% of the examined patients. However, no clear differentiation between periligamentous edema, partial or compleate tear was made. In the present study the reported injury rate for dITT lesions was 21.3%, with 8.0% beeing complete- and 13.3% beeing partial ruptures, respectively. In addition to any previous research, injury data for both structures within the same patient are reported. A combined lesion of both structures occurred in 12.7% of the patients, with 2.8% beeing partial- and 9.9% being complete ruptures. Compared to adults, a lower injury rate for both total- and partial lesions was observed in pediatric patients.

**Table 5:** MRI visibility of the deep attachment of the ITT

Authors	Year	No. of patients	Subjects	Anatomical definition	Identification	Injury	Interrater reliability (Kappa value)	Intrarater reliability (Kappa Value)
Khanna et al. [21]	2018	20	Patients with ACL tears and Pivot-shift bone-marrow-edema	Proximal and epicondylar fibers running from the ITB to the femur	85%	Proximal band: 82% Epicondylar band: 29%	n.s.	n.s.
van Dyck et al. [50]	2019	69	Patients with ACL surgery	Low signal intensity fibers attaching to the femur approximately 68 mm and 48 mm proximal to the femoral condyle	100%	No injury: 67% Mild periligamentous edema: 30% Partial rupture: 3%	0.922	n.s.
Batty et al. [2]	2019	50	ACL intact knees	Extra-articular, linear, posterolateral structure connecting the ITB to the femur	96% on sagittal view, 76% on axial view, 4% on coronal view	n.a.	Sagittal: 0.5 Coronal: 0.1 Axial: 0.2	n.s.
Liebensteiner et al. [28]	2020	71	Healthy knees without any major knee pathology	Deep attachments of the ITT with insertions near the septum intermuscolare, supracondylar or retrograde to the femur	60.6%	n.a.	0.94	0.94
Berthold et al. [3]	2020	10	Knee specimens	Proximal and distal fibers inserting distal to the lateral intermuscular septum at the metaphysis of the posterolateral femur	100% proximal fibers 90% distal fibers	n.a.	Excellent	Excellent
Marom et al. [32]	2020	72	Patients with acute ACL surgery	Discrete fibrous band with appropriate anatomic course and expected relationship to anatomic landmarks identified in at least 2 consecutive slices	82%/87%^a^	64%/71%^b^	Proximal fibers: 0.7 Distal fibers: 0.51	Proximal fibers: 0.89 Distal fibers: 0.66

*n.a.* not applicable, *n.s.* not stated, *ACL* Anterior Cruciate Ligament, *ITT* iliotibial tract, *ITB* iliotibial band

^a^Results of two separate reviewers; ^b^injury to Kaplan fiber complex (either proximal or distal or both Kaplan fibers)

Marom et al. [32] stated several potential factors being responsible for the differences in the rate of visualizing injuries to the dITT. Different MRI devices, MRI parameters and examination protocols as well as differences in the training and experience of examiners might influence outcomes when detecting lesions to the anterolateral ligamentous structures of the knee. In the present study PD-weighted fat satured axial and coronal images were preferred by both fellowship trained musculoskeletal radiologist for visualizing both the ALL and the dITT injuries.

The present data indicate that concomitant ALL or dITT injuries are common in ACL deficient knees; however, a combined lesion of both structures is rather rare. This information might be of special clinical relevance in the treatment of rotatory unstable knees, where an anatomical extra-articular, antero-lateral reconstruction is planned and, therefore, a preoperative in-depth understanding of the injured structures required.

This study has some limitations. First, this was a retrospective study bearing a selections bias. Second, MRI scans, where taken within the first 6 week post trauma. During this time, the hemorrhage surrounding the anterolateral soft tissue structures might have reabsorbed thus complicating the visualization. Moreover, there are no validated diagnostic criteria to detect lesions to the ALL and especially the dITT. To overcome this knowledge gap, general principles for diagnosing ligamentous and soft-tissue injuries were used. The major strengths of the present study were strict inclusion criteria, a large cohort of patients, the examination of pediatric patients, and the use of a modern 3-T MRI scanner. Additionally, all examinations were performed by two specialized and certified musculo-skeletal radiologists with more than 15 years of experience. Finally, the incidence as well as inter- and intra-observer reproducibility in detecting lesions of the ALL and dITT in the same patient was assessed for the first time.

Clinically, the preoperative visualization of potentially injured structures of the anterolateral knee is crucial and is important for a more personalized preoperative planning and tailored anatomical reconstruction. Further biomechanical and clinical studies are needed to better understand the impact of injuries to the anterolateral complex of the knee.

## Conclusion

In ACL injured knees, tears of the ALL (58.4%) are observed more frequently compared to lesions to the deep iliotibial tract, including the Kaplan fiber complex (21.3%). Combined injuries of both structures are rare. Injuries to the ALL and the deep portion of the ITT can be assessed with high inter- and intra-observer agreement using MRI.

## Abbreviations

ITT: Iliotibial tract
ALL: Anterolateral ligament
MRI: Magnetic resonance imaging
dITT: Deep portion of the iliotibial tract
